# Augmentation uretero-enterocystoplasty for refractory urinary tract dysfunction: a long-term retrospective study

**DOI:** 10.1186/s12894-021-00927-z

**Published:** 2021-11-30

**Authors:** Xiaoqian Ying, Limin Liao

**Affiliations:** Department of Urology, Beijing Boai Hospital, China Rehabilitation Research Centre; School of Rehabilitation of Capital Medical University, No 10. Jiaomen Beilu, Fengtai District, Beijing, 100068 China

**Keywords:** Augmentation cystoplasty, Refractory bladder dysfunction, Ureteral re-implantation, Efficacy, Complications

## Abstract

**Objectives:**

To report the long-term efficacy and complications of the augmentation uretero-enterocystoplasty (AUEC), including augmentation cystoplasty with simultaneous ureteroplasty and ureteral anti-reflux implantation in a single center.

**Methods:**

We retrospectively reviewed clinical records, video-urodynamic data, and magnetic resonance urography of 210 patients who underwent the procedure for refractory lower urinary tract dysfunction (LUTD) from 2003 to 2019. International vesicoureteral reflux (VUR) and upper urinary tract dilatation (UUTD) grading systems were applied to assess upper urinary tract function, and post-operative complications were assessed.

**Results:**

Mean age was 28.1 years, with a mean follow-up time of 57.4 months. A total of 338 ureters were simultaneously re-implanted, and ureteroplasty was performed on all ureters. There was a significant postoperative improvement in the bladder capacity, intravesical pressure, and compliance (*P* < 0.05). VUR improvement rate was 97.7% and postoperative improvement of UUTD presented in 72.5% ureters. Mean serum creatinine (Scr) level was significantly improved compared to preoperative Scr values (226.0 ± 89.4 μmol/L vs. 217.5 ± 133.9 umol/L, *P* < 0.05). The 1.0% patients had unacceptably postoperative urinary incontinence and 85.4% preoperative megaureters were improved. Primary complications included metabolic acidosis (9.5%), vesicoureteral anastomosis stenosis (6.2%), persistent VUR (2.7%), urinary calculi (6.6%), and intestinal dysfunction requiring laparotomy (3.3%).

**Conclusion:**

In the study, a large series of patients treated with a complex surgical procedure was reported. It is novel, as this case series represents patients with aggressive surgical correction of VUR, ureteral tortuosity and upper tract dilation at the time of AC. AUEC was shown to have a positive role in treating patients with refractory LUTD associated with hydronephrosis and ureteral dilatation, stenosis or obstruction, with or without high- or low-pressure VUR. It was effective in improving renal function and protecting the UUT function from further deterioration in most patients with renal insufficiency.

## Introduction

Augmentation cystoplasty (AC) was firstly performed in an experimental dog with ileum at the end of 19th century, then it was applied to the patients with neurogenic bladders and small tuberculous bladders [1]. Its aim is to increase bladder compliance and capacity, decrease bladder pressure, preserve renal function, and sustain urinary continence after the failure of conservative urinary management [2]. Various indications and substitution, and surgical techniques have been described in patients and animals with lower urinary tract dysfunction (LUTD) in recent decades [3]. Laparoscopic and robot-assisted procedure in AC showed feasible and safe outcomes as well [4]. Nowadays the gold standard for AC is enterocystoplasty. However, there is no consensus regarding simultaneous ureteral re-implantation (URI) and its efficacy. High intravesical pressure may result in vesicoureteral reflux (VUR), which is an important factor for upper urinary tract dilatation (UUTD), pyelonephritis, renal scarring, and renal deterioration [5]. It was indicated that more than 50% patients with high-grade VUR (grades III–V) underwent AC without simultaneous URI had residual high-grade VUR [6]. VUR initiated at lower intravesical pressure was suggested to be corrected concomitantly during AC as well [7]. Nevertheless, other studies have shown that performing AC alone could resolved VUR in most patients, thus URI is unnecessary [8].

To improve LUTD and protect kidneys from damage, we performed a surgical procedure that combines AC with simultaneous ureteroplasty and ureteral anti-reflux implantation (UARI), which we called augmentation uretero-enterocystoplasty (AUEC). To describe and analyze the safety and efficacy of the procedure with simultaneous UARI, we carried out the retrospective study.

## Materials and methods

### Subjects and assessment

This is a retrospective case series, from the urological surgeons leading by Limin Liao at China Rehabilitation Research Center. After obtaining Institutional Review Board approval from our center, we retrospectively reviewed the medical records of total patients underwent AUEC from 2003 to 2019. Preoperative anti-muscarinic administration and clean intermittent catheterization (CIC) worked poorly in all these patients. Some cases also adopted other therapies. Eight patients (3.8%) failed to manage urinary tract symptoms via botulinum toxin A (BTX-A), five patients (2.4%) underwent sacral neuromodulation, nineteen patients (9.1%) in ureteral stenting, five patients (2.4%) suffered ureteral re-implantation without AC, and 2 patients (1%) underwent somatic-visceral nerve reconnection procedure. Five patients (2.4%) had suffered the unilateral kidney resection. Most patients had decreased bladder capacity, low compliance, high intravesical pressure, and normal or high urethral pressure. All video-urodynamics (VUD) studies, magnetic resonance urography (MRU), and serum creatinine (Scr) levels were analyzed. VUD studies were performed according to Good Urodynamics Practice [9] with a filling rate of 10 ml/min. Ureteral dilatation and hydronephrosis was evaluated according to MRU-UUTD system described by Liao [10, 11] and VUR was graded with the international grading system for vesicoureteral reflux [12]. The Scr level was derived from hematologic examination. Ureteral obstruction or stenosis were identified by radioactive renogram and MRU. Complications were recorded via medical records or telephone inquiries. MRU in 39 of 210 cases lost to follow-up, and no VUDS in 29 cases. All patients’ Scr values and postop complications was obtained.

### Surgical indications

To undergo AC, patients had to have at least one of the following conditions: (1) detrusor overactivity (DO) with high intravesical pressure (> 40 cm H2O) during urinary storage phrase or lower bladder compliance (< 10 ml/cm H_2_O); (2) socially unacceptable urinary incontinence due to DO or severely decreased bladder capacity; (3) high-grade and/or low-pressure VUR with UUT deterioration; (4) high-grade UUTD with UUT deterioration; (5) infective or inflammatory disorders (e.g., tuberculous bladder contracture); or (6) a significant decrease in the Scr level (> 1.5 mg/dL [132.6 umol/l]) after indwelling urethral catheterization in patients with chronic renal failure.

The indications for UARI during AC included at least one of the following conditions: (1) high-grade VUR (grade III–V) during urinary storage phrase [13]; (2) VUR at low intravesical pressures (< 10 cm H_2_O); and (3) high-graded UUTD (grade 3–4) according to MRU-UUTD system combined with ureteral tortuosity; (4) vesico-ureteral junction (UVJ) stenosis. The indication for ureteroplasty (ureterolysis and tailoring/shortening) during AUEC included megaureter, severely tortuous ureter, and stenosing ureteric stenosis.

### Technique

**Fig. 1:**
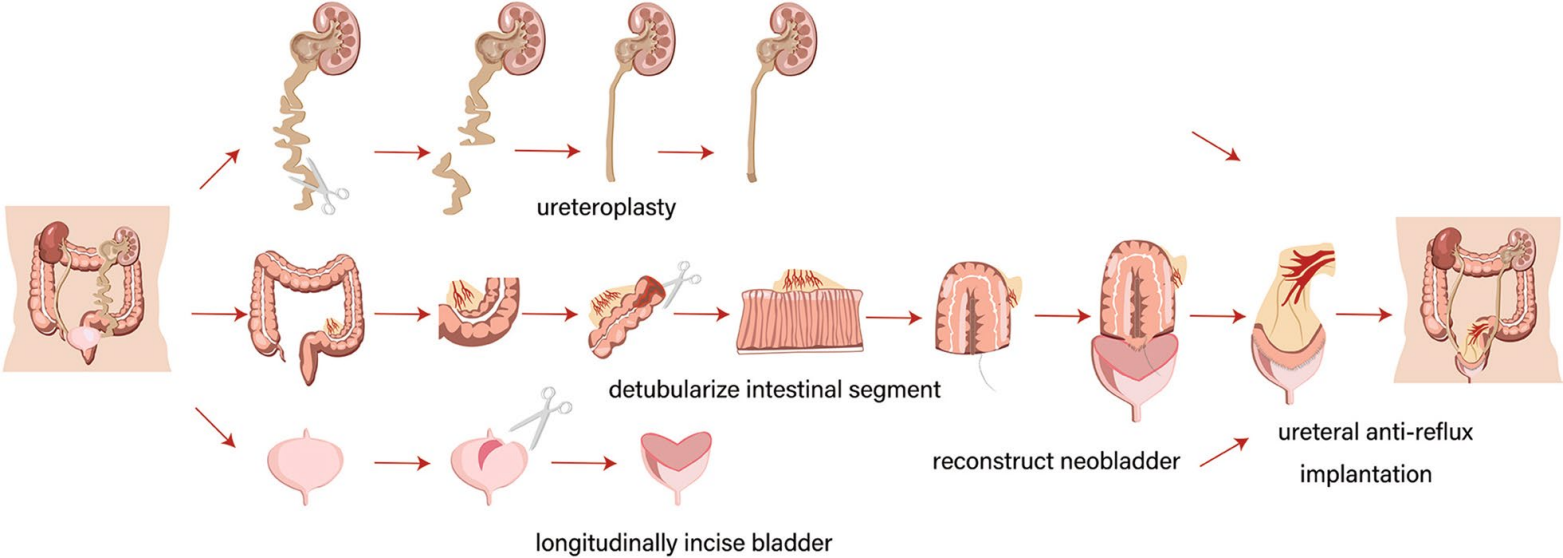
Schematic diagram for AUEC involving the unilateral ureter (The copyright of Figure is attributed to Limin Liao. The manuscript has got permissions to use the image)

AUEC with concomitant unilateral ureter treatment was performed with patients in the supine position (Fig. 1). We identified and detached the affected ureter from UVJ. About 25 cm sigmoid was isolated as a substitution.

The ends of the original intestine are anastomosed end-to-end. Anhydrous alcohol was used to deal with the isolated intestinal segment. The segment was detubularized along the border of the mesentery and it was sutured in a “U” (sigmoid) [14] shape to form a substitutable patch.

Urinary bladder was longitudinally incised and megaureters, severe tortuous ureters, or stenosing ureteric stenoses were performed simultaneous ureteroplasty, including ureterolysis and tailoring/shortening. Ureterolysis refers to mobilization and straightening of the ureter. Tailoring refers to shortening the length of the ureter, and reducing the diameter of the megaureters, including ureterolysis and tailoring/shortening. Ureterolysis refers to mobilization and straightening of the ureter. Tailoring refers to shortening the length of the ureter, and reducing the diameter of the megaureters. We made a hemi-Kock nipple valve with original ureter, and the plastic ureter was re-implanted on the native bladder or bowel depend on the fibrotic tissue and the thickness of bladder wall, and bladder contracture. Double-J catheters were inserted for postoperative urinary drainage. A suprapubic catheter and two tubes were respectively placed for the postoperative neobladder, intra-abdominal and retropubic drainage. If the Mitrofanoff procedure was acceptable, it can be performed simultaneously.

### Statistical analysis

Quantitative data are presented as the mean ± SD. The paired Student's t-test was used to compare preoperative with postoperative values. SPSS 21.0 was used, and a *P* < 0.05 was considered statistically significant.

## Results

### Patient characteristics

**Fig. 2:**
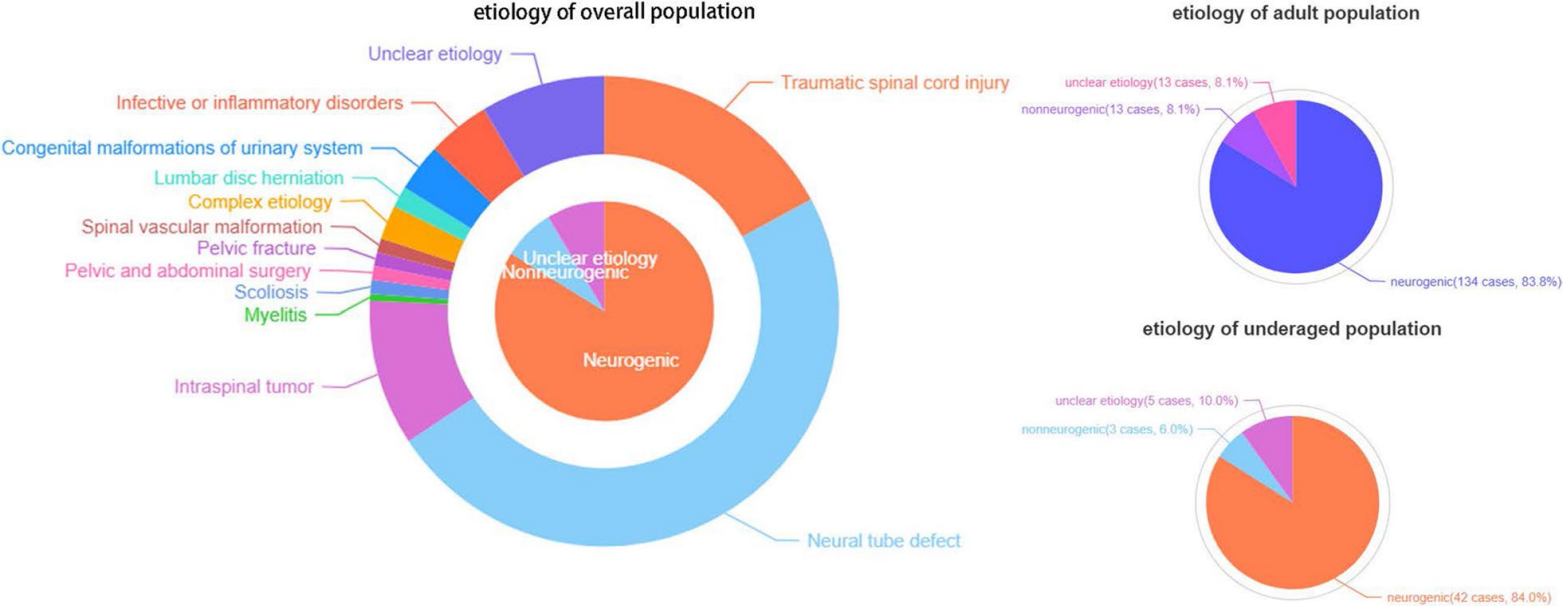
Etiological characteristics of 210 patients

Our study involved 153 males (72.9%) and 57 females (27.1%), and the mean age of these patients was 28.1 years (range: 4–67 years). The mean duration of their lower urinary tract symptoms was 13.5 years (range: 1–56 years). The mean follow-up was 57.4 months (range:1–151 months). Table 1 and Fig. 2 listed the etiologies of their clinical diagnoses.

**Table 1:** Clinical diagnosis of 210 patients

Clinical diagnosis	No. Pts (%)
Neurogenic	176 (83.8)
Traumatic spinal cord injury	36 (17.1)
Neural tube defect*	102 (48.6)
Intraspinal tumor	21 (10.0)
Myelitis	1 (0.5)
Scoliosis	2 (1.0)
Pelvic and abdominal surgery	2 (1.0)
Pelvic fracture	2 (1.0)
Spinal vascular malformation	2 (1.0)
Complex etiology**	5 (2.4)
Lumbar disc herniation	3 (1.4)
Nonneurogenic	16 (7.6)
Congenital malformations of urinary system developmental defect	7 (3.3)
Infective or inflammatory disorders	9 (4.3)
Unclear etiology	18 (8.6)
Total	210

*Including myelomeningocele, spina bifida and tethered cord syndrome

**Complex etiology ≥ two clinical diagnoses

### Preoperative and postoperative VUD

Postoperative detrusor pressure decreased significantly (*P* = 0.001), and a significant increase presented in postoperative bladder capacity and compliance compared to preoperative VUD parameters: mean detrusor pressure was decreased from 36.0 ± 28.0 cm H_2_O to 17.2 ± 15.0 cm H_2_O (*P* = 0.0001); mean bladder capacity was increased from 220.8 ± 168.4 ml to 443.1 ± 161.2 ml (*P* = 0.001); and compliance was increased from 8.9 ± 11.1 ml/cmH_2_O to 42.7 ± 62.9 ml/cmH_2_O (*P* = 0.001). Mean intravesical pressure was decreased from 44.1 ± 26.3 cm H_2_O to 24.5 ± 15.8 cm H_2_O (*P* = 0.042).

### Hydronephrosis and ureteral dilatation improvement

We totally performed simultaneous UARI on 338 ureters during AC, and ureterolysis and/or cutting was performed on all ureters. According to MRU-UUTD system, significantly postoperative improvement of UUTD presented in 245 ureter units (72.5%), unchanged UUTD was observed in 66 ureter units (19.5%), and deterioration in 27 ureter units (8.0%).

### Vesicoureteral reflux and improvement

Preoperative VUR was detected in 175 ureter units. According to postoperative VUD, residual VUR was observed in only 4 ureter units (2.3%), and the UARI improvement rate was as high as 97.7% (171 ureter units).

### Renal function changes

Fifty-nine LUTD patients reported preoperative chronic renal insufficiency, and 74.6% of them had postoperative improvement according to the latest checkups. Mean Scr level was significantly improved compared to preoperative Scr values (226.0 ± 89.4 μmol/L vs. 217.5 ± 133.9 μmol/L, *P* = 0.033). Two (1.0%) patients with preoperative chronic renal insufficiency and required dialysis after 3 years, and a patient diagnosed with new onset uremia.

### Urinary incontinence and megaureters

Before the operations, all these patients were diagnosed as varying degree of urinary incontinence and two patients (1.0%) had unacceptably postoperative urinary incontinence. They were treated with artificial urethral sphincter (AUS) implantation after AUEC. Totally 219 preoperative megaureters were observed and they were improved with a rate of 85.4% (187 ureters).

### Complications

#### Metabolic acidosis

Twenty cases (9.5%) developed metabolic acidosis (CO_2_ combining power < 22 mmol/L) accompanied by abnormally increased Scr values and serum chlorine (> 110 mmol/L). Only 8 (2.2%) cases reported new-onset postoperative metabolic acidosis among them. These patients recovered uneventfully after the oral bicarbonate or infusion administration.

#### Stenotic vesicoureteral anastomosis

Preoperative ureteral obstruction was detected in 179 ureters (53.0%). A postoperative vesicoureteral anastomotic stricture (VUAS) was observed in 14 ureters (4.1%) of 13 patients (6.2%). One of these cases had a large number of inflammatory polyps which obstructed the anastomosis of the bilateral ureteral orifices. Unilateral VUAS in the other 12 cases (3.6%). Among them, VUAS in 9 (2.7%) ureters was residual for incompletely solved obstruction and new onset VUAS in 5 (1.5%) ureters. The treatment included UARI again for 4 cases, the placement of D-J catheters or stents in 9 cases. Finally, VUAS in 12 ureters were improved apparently after above interventions. UUTD persisted for residual VUAS in 2 ureters of 2 cases and percutaneous nephrostomy was performed for.

#### Vesicoureteral reflux

There were 9 ureters (2.7%) with VUR within 1 year after the procedure, including residual VUR in 4 ureters (1.2%) and new-onset VUR in 5 ureters (1.5%). All these VUR was initiated at an intravesical pressure lower than 40 cm H_2_O. After the treatment with anti-muscarinics and antibiotics or watchful waiting, VUR in 4 ureters solved and VUR in 5 ureters (1.5%) persisted at the final evaluation.

#### Urinary tract infection and stone formation

Preoperatively recurrent symptomatic urinary tract infection (UTI) was reported in 97 (46.2%) cases. Cases suffered postoperative symptomatic UTI is less than 20%, and 3 (1.4%) cases were diagnosed as epididymitis. Urinary calculi occurred to 14 (6.6%) cases, including bladder calculi in 6 cases (2.9%) and ureteral or renal calculi in 8 cases (3.8%). Ten cases were treated with endoscopic lithotripsy or removal and recovered uneventfully, and the other 4 cases were still under watchful waiting.

#### Bowel dysfunction and malignancy

Postoperative intestinal obstruction occurred in 7 (3.3%) cases. They were treated with laparotomy and recovered without any events. Changes in bowel habits were reported in 10 (4.7%) cases, including fecal incontinence in a case (0.5%), diarrhea in 6 (2.9%) cases and improved constipation in 3 (1.4%) cases. Malignancy in neobladder was not detected during follow up.

## Discussion

Due to similar proportions of etiology (adults vs. underage: neurogenic 83.8% vs. 84.0%; nonneurogenic 8.1% vs. 6.0%; unclear 8.1% vs. 10.0%; Fig. 2), we analyzed data from adult and underage patients in combination. In the study, male patients were more than females and most patients suffered neurogenic bladder.

Patients with neurogenic bladder are at increased risk for the storage and emptying of bladder, hydronephrosis, and renal function deterioration [15]. According to the study of Adam and his colleagues, UUT alternations and renal damages was at low incidence within 8–11 years for multiple aggressive medical management [16]. As a surgical intervention, AC works to create a hypo-pressure storage reservoir, which facilitates increasing bladder capacity and compliance, protect against renal function damages, improving urinary continence and the quality of life. Various human tissues sources and synthetic materials in AC have been described, including bowel, stomach, ureters, peritoneum, omentum, polyvinyl sponge, gelatin sponge, collagen with polyglactin membrane and so on [17–19]. Due to the incidence of their complications, enterocystoplasty is the preferable choice and the commonly patches are from ileum and sigmoid. We preferred to use sigmoid segments for the large intestinal cavity, rich mesentery, thick intestinal wall, and available maneuverability [20]. The choice, however, comes with the disadvantages of numerous mucus secretion, high occurrence of urinary systems infections and stone formation. In the longer study, malignancy was reported as well. Ileum was adapted for being easily handled, abundant mesentery and blood supply. However, metabolic disturbances, significant anemia and bowel obstruction were common.

Simultaneously, URAI were performed with AC in these patients according to our indications. In addition to the significantly improvement in postoperative bladder capacity, compliance and intravesical pressure, 97.7% VUR and 72.5% UUTD improvement was observed. Postoperatively ameliorated renal function presented in 74.6% of patients with preoperative chronic renal insufficiency. AC alone plays a positive role in improving urodynamics, but the function of URI or URAI was controversial. VUR was considered as a crucial factor for complications of upper UTI and renal damages [21]. It was turned out that 0–47.3% of VUR was residual after AC alone [6, 7]. Simultaneous URAI could decrease the incidence to 4% [22]. In our study, only 2.3% residual VURs was observed. Some researchers suggested VUR was secondary to hypo-compliance and high intravesical pressure, thus VUR could be solved after AC alone. However, VUR at the low intravesical pressure made it less stringency, which was assumed relevant to ureterotrigonal insufficiency [7]. On the other hand, URAI was not encouraged to be performed in significant bladder contracture for increased technical difficulties and the risk of postoperative VUAS or obstruction from thickening or fibrosis of the bladder wall [23]. To avoid these risks, we commonly chose to perform URAI in the soft bowel patches fixed on the neobladder. Most urinary incontinence and megaureters was improved after AUEC. Merely unacceptable urinary incontinence was reported (1%), which was tightly relevant to low urethral closure pressure in the study. During AUEC, 5 cases (2.4%) had catheterisable channels created with appendix and ileum at the same time.

Commonly, complications were primarily associated with the sources of patches. Metabolic abnormalities were usually derived the usage of various patches. Reabsorption and secretion of bowel patches was the general underlying mechanism for electrolyte disturbances [24]. In other study, more than 15% patients were treated with oral bicarbonate for metabolic acidosis while it was less than 10% at our center. Baseline normal renal function of most patients may play a positive role for the outcomes. Postoperative VUAS was a structural complication, which often resulted from surgical techniques, the site URAI was performed and the perioperative local mucomembranous edema according to our experience. Timely individual interventions could solve it uneventfully. The postoperative VUR within 12 months was observed in other study as well [7]. It was hypothesized that augmented bowel need time to became expanded and more compliant in the early months after the procedure.

Symptomatic UTI in 22.7% of patients underwent AC with an ileal and 8% in patients suffered a gastrocystoplasty had been reported [25]. Symptomatic UTI included positive urine routine test and getting febrile in the article. More than 50% recurrent symptomatic UTI was solved after AUEC. About 6–52% patients underwent enterocystoplasty was detected with postoperative urinary calculi [26, 27]. Insufficient bladder emptying and urinary stasis increased the risks of urinary stone formation. Furthermore, bacteria, mucus secreted by bowel patches and metabolic abnormalities were very relevant to stone formation. To decrease the risks of UTI and stone formation, we administrated these patients with sufficient CIC and routine bladder irrigation.

The postoperative intestinal obstruction was 3.3%, which was similar with the reported data (3%-3.2%). As a fatal complication, spontaneous bladder perforation was not observed. Avoiding technical error and careful suture and fixation during the procedure was our experience. Although urinary bladder cancer has been reported, we didn’t detect malignancy in these patients [28]. We presumed that 90% tumors were diagnosed after 10 years [29] and the follow-up time over 10 years of these patients was rare (3 cases, 1.4%). In the study, we were mainly aimed to recognize and report the effects of AUEC in all LUTD patients based our indications, no matter what causes were. Therefore, a small number of patients with nonneurogenic and unclear etiology were included in analysis. Generally, AUEC or AC for refractory LUTD resulting from various etiology (especially in tuberculous cystitis or urinary tract tuberculosis) was likely considered to produce different outcomes. However, the comparison between urinary tract tuberculosis causes and other etiology was rarely reported. A long term follow-up study (mean postoperative follow-up 11.1 ± 9.1 years) of de Figueiredo and colleagues suggested tuberculous cystitis patients obtained good bladder capacity, good compliance and normal sensation after AC with or without URI using detubularized sigmoid segments [30]. And preoperative renal function maintained in 80% cases. An Indian center also indicated AC with or without URI reconstruction could increases the bladder capacity and preserves UUTs [31]. In our study, nine cases underwent AUEC for indications resulting from inflammatory disorders. Of these cases, eight (88.9%) maintained preoperative renal function or got improvement. A case (11.1%) come into bad results for postoperative vesicoureteral anastomotic obstruction from bladder inflammatory polyps, although VUD parameters improvement had been observed. Mitrofanoff procedure was rarely performed in our center. Additionally, postoperative AUS implantation was tightly linked with originally low urethral pressure.

Whatever it goes, preoperative estimations for the capacity of CIC and long-term dynamic evaluations are still very important for patients treated with the procedure. In younger patients, CIC is performed by the nurses or their parents. The volume of CIC referred to the maximum bladder capacity from VUD.

## Conclusions

We reported a large series of patients treated with a complex surgical procedure (AUEC). It is novel, as this case series represents patients with aggressive surgical correction of VUR, ureteral tortuosity and upper tract dilation at the time of AC. The results showed that AUEC procedure is safe and effective for most patients with refractory LUTD associated with hydronephrosis and ureteral dilatation, stenosis or obstruction, with or without high- or low-pressure VUR. It extends the indications for the AC. This technique played a positive role in stabilizing renal function and protecting the UUT function and residue renal function from further deterioration in most patients with renal insufficiency.

## Data Availability

All the data and materials are available if it’s for reasonable request. Please contact corresponding author Limin Liao if the data was requested.

## Abbreviations

AC: Augmentation cystoplasty
AUEC: Augmentation uretero-enterocystoplasty
AUS: Urethral sphincter
BTX-A: Botulinum toxin A
CIC: Clean intermittent catheterization
DO: Detrusor overactivity
LUTD: Lower urinary tract dysfunction
MRU: Magnetic resonance urography
Scr: Serum creatinine
URAI: Ureteral anti-reflux implantation
URI: Ureteral reimplantation
UTI: Urinary tract infection
UUTD: Upper urinary tract dilatation
UVJ: Vesico-ureteral junction
VUD: Video-urodynamics
VUAS: Vesicoureteral anastomotic stricture
VUR: Vesicoureteral reflux
