# Correction to: Augmentation uretero-enterocystoplasty for refractory urinary tract dysfunction: a long-term retrospective study

**DOI:** 10.1186/s12894-022-00971-3

**Published:** 2022-02-16

**Authors:** Xiaoqian Ying, Limin Liao

**Affiliations:** Department of Urology, Beijing Boai Hospital, China Rehabilitation Research Centre, School of Rehabilitation of Capital Medical University, No10. Jiaomen Beilu, Fengtai District, Beijing, 100068 China

## Correction to: BMC Urology (2021) 21:166 https://doi.org/10.1186/s12894-021-00927-z

Following publication of the original article [1], the authors would like to correct the affiliation information for authors. It has been corrected in this correction.

1. Department of Urology, Beijing Boai Hospital, China Rehabilitation Research Centre; School of Rehabilitation of Capital Medical University; No10. Jiaomen Beilu, Fengtai District, Beijing 100068, China.
2. “Department of Urology of Capital Medical University, Beijing 100068, China”, should be removed.

The original article [1] has been corrected.
